# Attentional decoupling while pursuing intentions: a form of mind wandering?

**DOI:** 10.3389/fpsyg.2013.00693

**Published:** 2013-10-01

**Authors:** Anna-Lisa Cohen

**Affiliations:** Department of Psychology, Yeshiva University (NYC)New York, NY, USA

**Keywords:** prospective memory, memory for intentions, managing attention, ongoing task costs, mind wandering

## Abstract

In the current study, participants performed an ongoing lexical decision task (LDT) in which they had to classify letter strings as words or non-words. In intention conditions, they also had to encode a postponed intention to remember to make a different response if a pre-specified cue appeared. Attempting to replicate an important finding from Cohen et al. (2008), the interest was in examining how varying cognitive load associated with an intention influences attention to the ongoing task (measured by reaction times). Typically, disengaging from a primary task is perceived as negative as it can lead to performance decrements, however, if disengaging from a primary task helps one to accomplish a desired future goal, then these attentional shifts may in fact be constructive. Results replicated those of Cohen et al. (2008) and showed that participants were very flexible in how they managed attention in the ongoing LDT. Reaction time costs emerged when cognitive load was high and solely for word trials (i.e., not for non-word trials). The implications for mind wandering are that, while our attention may wander when stimuli are present that trigger a suspended or unfulfilled goal, we are better able to stay on task when the stimuli are less goal relevant. Therefore, the decoupling process (e.g., Schooler et al., 2011) might be initiated when postponed goals are accompanied by a high degree of cognitive load and when external stimuli are present that relate to that goal.

## Introduction

In everyday life, people strive to attain multiple goals, some that are simple and short-term (e.g., choosing a shampoo brand), and others that are complex, long-term and require repeated efforts (e.g., applying for jobs). The average individual must consider viable opportunities to act on suspended goals while maintaining high levels of performance on ongoing behaviors. Very often, goals must be postponed until the appropriate moment arrives for their execution. Prospective memory (PM) refers to remembering to carry out delayed intentions (Einstein and McDaniel, 1990). The successful execution of intentions has important implications for many facets of everyday life including health (remembering to take medication), social (remembering a friend's birthday), and safety (remembering to turn off the stove before leaving the house).

Intention completion requires that the cognitive system be configured in such a way that the person is sensitive to information that facilitates a goal-relevant behavior or encodes a sufficient link between an anticipated environmental cue and an action (Gollwitzer, 1993, 1999). Indeed, some research (e.g., Goschke and Kuhl, 1993; Marsh et al., 1998; Cohen et al., 2005, 2011) has shown that information related to intentions is highly accessible compared to information that is not future-oriented. This phenomenon was termed the “intention superiority effect” (Goschke and Kuhl, 1993). For example, if a person must remember to mail an important letter, he or she might be especially sensitive to noticing a mailbox or any stimulus that relates to posting a letter while performing an unrelated activity such as walking the dog. Noticing a stimulus that is relevant to a postponed goal involves a shift of attention from a currently active goal (e.g., walking the dog) to a previously encoded goal (e.g., to mail a letter). A thematically similar hypothesis was advanced by (Klinger, 1975, 1977, 2009, and most recently, Klinger and Cox, 2011). According to Klinger's Goal Theory of Current Concerns, selective attention is largely determined by a person's current concerns. Current concerns are cognitive-affective motivational states that are activated once a person commits to a goal and remain active until either the goal is achieved or abandoned (Klinger and Cox, 2011).

In typical laboratory studies of PM, participants perform an ongoing task in which they are presented with a series of stimuli requiring some classification [e.g., making word/non-word judgments in a lexical decision task (LDT)]. In PM conditions, participants are provided with an additional instruction to respond differently if a particular target event happens in the future (e.g., “Press F1 if the word FLOWER appears”). Thus, these paradigms are sensitive to participants' ability to encode an intended future action and to act on that intention at the appropriate time. It is important to note that participants are not instructed to try to think about the delayed intention while performing the ongoing primary task. That is, participants are simply given an intention to carry out but they do not receive specific instruction regarding how (or whether) they should allocate attention to the PM task. Indeed, participants are clearly instructed at the beginning of the experiment that their primary objective is to perform the LDT as quickly and accurately as possible. The PM instructions tend to be delivered in such a way that participants are led to believe that the cues might occur in the subsequent block of trials, though there is also a possibility that the cues will not appear. In this context, thinking about the delayed intention while performing the ongoing task is actually counterproductive to the primary task objective which forms the majority of trials. Therefore, when the mind wanders to thoughts about the intention while performing the LDT, this attentional decoupling is similar to states such as daydreaming or absented minded lapses. In these instances, consideration of the primary task is reduced in favor of the active consideration of internally generated thoughts related to the PM task. Much research has demonstrated that PM tasks lead to interference costs in ongoing task performance (e.g., Smith, 2003; Einstein et al., 2005; Hicks et al., 2005; Cohen et al., 2012; but see Einstein et al., 2005; Scullin et al., 2009; for exceptions). These interference costs are thought to occur because processing of the ongoing task breaks down in favor of thoughts related to the intention. In Cohen et al. (2008), participants performed an ongoing LDT in which they were instructed to respond as quickly and accurately as possible. Then, halfway through the task, participants were given a PM task, specifically, to remember to make a different response (press the F1 key) if 1, 2, 3, 4, 5, or 6 pre-specified cues appeared during the LDT. Results revealed a linear trend in which there was no significant increase in LDT latencies with one or two PM targets but significant costs did emerge with three or more targets. Therefore, in conditions of low cognitive load, the delayed intention could be maintained in mind with no observable costs to the primary task.

An important goal of the current paper was to replicate the response time pattern from Cohen et al. (2008). In the Cohen et al. (2008) study, accuracy data in the LDT was not recorded and therefore not reported; Smith (2010) suggested that due to the absence of accuracy data, response time data could not be clearly interpreted. In Cohen et al. (2008), there were no ongoing task costs when participants had one intention-related cue. Smith (2010) proposed that this lack of ongoing task costs in the one-word condition might have been due to sacrificing speed for accuracy in the baseline block, leading to a greater speed increase in Block 2, thereby canceling out the possibility of observing costs in the one-word cue condition. This alternative interpretation could not be satisfactorily addressed in Cohen et al. (2008) since accuracy on the LDT was not reported. Therefore, a goal of this study was to analyze accuracy data so that Smith's (2010) interpretation could be properly examined.

Replicating findings from Cohen et al. (2008) allowed us to investigate the potentially illuminative similarities and differences between PM and mind wandering. In a typical mind wandering paradigm, participants perform a primary task such as reading a passage and then report on the number of times that attention wanders spontaneously to thoughts unrelated to the ongoing task (e.g., Schooler et al., 2004; Smallwood et al., 2008; Smallwood, 2011). In PM tasks, participants receive instructions for an ongoing task (e.g., reading comprehension task) and must also make a different response if a pre-specified intention-related target event occurs. In a PM task paradigm, intention-related thoughts that come to mind are comparable to mind wandering because they cause attention to shift away from the primary task. Participants must rely on their own strategies as to how they juggle the primary task demands while also maintaining the intention. In this PM context, thinking about the delayed intention while performing the ongoing task is analogous to mind wandering in the sense that it is actually counterproductive to the primary task objective of performing well on the reading comprehension task. Participants must strike a balance between devoting attention to the primary task while not forgetting to execute the delayed intention. Successful performance of a PM task involves periodic shifts away from the primary task to thoughts about a previously encoded intention. When participants are given a PM task, they are not instructed to divert attention away from the primary task. However, much like in mind wandering episodes, intervening thoughts about an impending intention led to performance costs very similar to those produced during mind wandering episodes.

Typically, mind wandering is thought to lead to performance decrements, however, if disengaging from a primary task helps to accomplish a desired future goal then mind wandering can be constructive. If mind wandering helps one to advance toward a desired goal, then mind wandering (or interference, in a PM paradigm) can be thought of as “functional.” This idea of “functional mind wandering” has been discussed by others (e.g., Klinger, 2009; Baird et al., 2011; Schooler et al., 2011) who suggest that mind wandering can be of service in completing prospective goals. As Klinger (2009) states, daydreams allow us to perform important and central functions in our life by reminding us of the details of our agenda. The interference costs in PM paradigms may be an example of this special case of functional mind wandering. Of course, the functionality of PM costs or mind wandering depends on the perceived value of the ongoing activity and the future goal/intention. For example, a professor disengaging from teaching his class to think about an upcoming golf game would not be functional, whereas a professor disengaging to think about taking his heart medication would be.

### Current study

In the current study, an objective was to replicate findings from Cohen et al. (2008) while also examining how these data inform us about mind wandering and the nuanced way that attention shifts between ongoing activity and unresolved goals. Participants performed one block of a LDT as a baseline in which they were instructed to respond as quickly and accurately as possible to letter strings. Then, halfway through the task, participants were given a PM task in which they had to make a different response if 1, 2, 3, 4, 5, or 6 intention-related cues appeared during the LDT. The interest was in determining how attention devoted to the primary LDT might vary according to cognitive load. Evidence from Cohen et al. (2012) demonstrated material specific costs such that reaction time costs to ongoing task performance emerged only on trials where the stimuli matched those of the intention-related target. Therefore, reaction time responses in the current study were analyzed separately for words and non-words trials in the LDT.

## Method

### Participants and design

A total of 136 Yeshiva University undergraduates volunteered to participate in the experiment in exchange for course credit. Each participant was tested individually in sessions that lasted ~30 min. The design was a 7 (Condition: control, one, two, three, four, five, or six targets) × 2 (LDT trial type: word, non-word) × 2 (Block: Block 1, Block 2) mixed factorial design with condition as a between-subjects factor and block (Block 1 or 2) and LDT trial type as within-subject factors.

### Materials and procedure

The protocol for this experiment was identical to Cohen et al. (2008). Participants were tested on a Dell laptop computer (Latitude/E6410). The experiment was performed using Presentation software (Version 0.70, www.neurobs.com). The six critical words used in this experiment had a medium level of frequency and were chosen from the Kucera and Francis (1967) norms. The lists in each block were matched with respect to frequency (mean frequency of 135 for both blocks), word length, and first letter. Non-words were created by moving the first syllable of each word to the end of each of the 126 total words (Hunt and Toth, 1990). The order of appearance was random for all string types. Participants made a total of 504 lexical decisions and a PM target appeared approximately every 20th trial, however, the number of intervening trials varied between targets so that participants could not predict when they would occur.

#### Phase 1

During phase 1, participants completed a consent form, after which instructions describing the experiment appeared on the computer screen. The instructions regarding the LDT explained to participants that letter strings would appear one at a time on the computer screen and that participants had to decide whether the letter string formed a word or a non-word. The instructions were worded very carefully to emphasize that participants should weigh accuracy and speed equivalently. Participants were then asked to position each index finger on the “F” and “J” keys and to press “F” if the string on the screen was a word and “J” if it was not a word, or vice versa (assignment of the computer keys “F” and “J” to words and non-words was counterbalanced across participants). Participants performed a first block of lexical decision trials consisting of 126 word trials and 126 non-word trials (252 in total). After the first block of trials, participants received instructions for the second portion of the LDT as well as for the embedded intention.

#### Phase 2

After the first block of LDT trials, participants received different instructions depending on the condition to which they were randomly assigned. Participants in the control condition were given a retrospective memory task in which they were asked to memorize six target words. They were told that they would have to recall the six words and the associated response (pressing the F1 key) at the end of the experiment as a memory check. This task served as the control condition because the participants assigned to it memorized words similar to the words in intention conditions but did not have to monitor for PM cues during the LDT component of the experiment. In other words, the PM component of the task (i.e., detecting cues) was eliminated. Participants who were randomly assigned to intention conditions were instructed to press the F1 key on the computer keyboard (after first making their lexical decision) if they saw any one of the PM targets during the experiment. For participants in all conditions, we emphasized the importance of their performance on the LDT and participants were encouraged to respond as quickly and accurately as possible with their word/non-word decisions. Of the participants in the intention condition, those in the one word condition had 30 s to memorize one word, and this target occurred 12 times during the second block of trials. Participants in the two-word condition had 30 s to memorize two words, and these targets occurred 6 times each during the second block of trials, for a total of 12 target appearances. In the three-word condition, participants had 60 s to memorize three words, and each of the three targets appeared 4 times in the second block for a total of 12 target occurrences. In the four-word condition, participants had 60 s to memorize the words, and each word occurred 3 times in the second block for a total of 12 target appearances. In the five-word condition, participants had 75 s to learn the five words and, since 12 is not divisible by 5, five of the words appeared twice (for a total of 10 target appearances) and two of the words then appeared 2 more times (for a total of 12 target appearances). We counterbalanced across participants which two words appeared an extra 2 times. In the six-word condition, participants had 120 s to learn the words, and each of the six words appeared twice, for a total of 12 target appearances. In all conditions, participants had to learn the words to criterion level, and the experiment did not proceed until participants demonstrated perfect recall. If recall of the PM targets was not perfect in any condition, participants were given 2 additional min and learning cycled through the same study–test procedure until it was perfect. PM targets occurred 12 times for every condition (with the exception of the control condition); therefore, we could isolate the manipulation of cognitive load. A PM response was deemed correct if it was made at any point before a word/non-word of the next trial was on the screen.

As previously mentioned, participants were instructed to make their lexical decision on each trial before making a PM response. This aspect of the design allowed participants to avoid the need to withhold their lexical decision response because they were trying to decide whether the word was a PM target. After each lexical decision keypress, participants were told that they could make their PM response (by pressing “F1”) before a word/non-word of the next trial was on the screen. The experimenter explained that on each trial the message “Press the space bar” would appear, indicating that participants should press the space bar with one of their thumbs to initiate the next trial. After reading the instructions, participants were asked to describe the instructions to the experimenter (to check their comprehension). They were then asked if they had any questions. After questions (if any) were answered, participants completed the two blocks of the experiment. Upon completion of the experiment, a post-experiment questionnaire was administered to test participants' recall of the PM target items and the associated action (pressing “F1”).

## Results

An alpha level of 0.05 was used in all analyses unless otherwise specified. At the end of the experiment, participants were asked to recall the PM targets as a memory check. Post-experiment recall of the PM targets was 100% recall for the one- through three-word conditions. Participants also had good recall for the control, four-, five-, and six-word conditions (control: *M*= 5.09 out of 6; four-word: *M* = 3.87 out of 4; five-word: *M* = 4.69 out of 5; six-word: *M* = 5.52 out of 6). There was no significant difference between conditions (*p* = 0.36). All participants recalled the associated action of pressing “F1.”

### Prospective memory task

**Figure 1:**
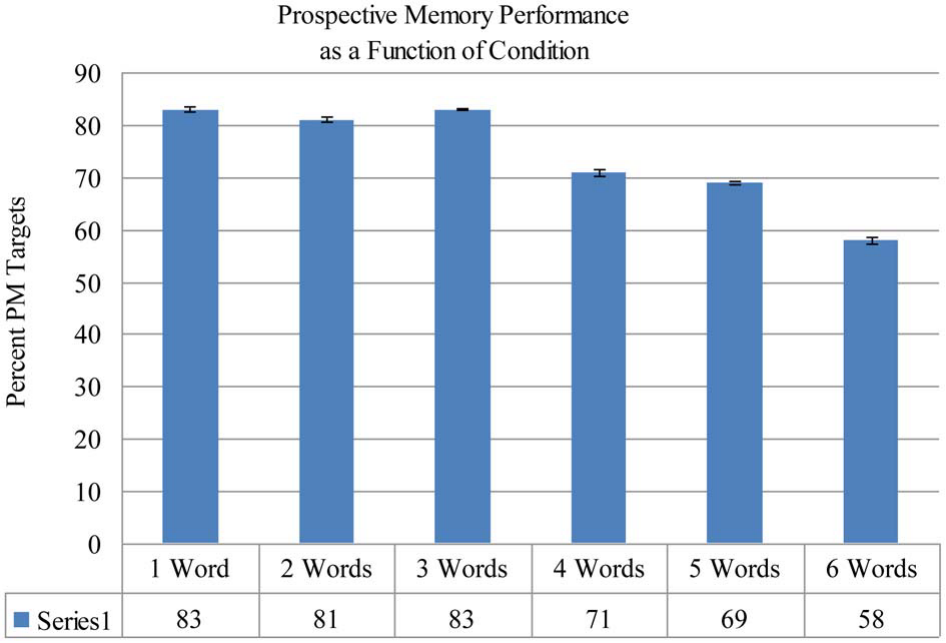
**Percent of prospective memory targets detected as a function of condition**. Bars represent standard error.

The proportion of PM targets correctly detected as a function of condition (number of PM targets: 1, 2, 3, 4, 5, 6) was submitted to a One-Way analysis of variance (ANOVA). In contrast to findings from Cohen et al. (2008), results yielded a significant effect of condition, *F*_(5, 115)_ = 5.02, *p* < 0.001, η^2^ = 0.18. Planned pair-wise comparisons revealed a number of significant differences. PM performance in the 1-word condition (83% correct) differed significantly from performance in the 5-word (69% correct) and 6-word (58% correct) conditions (all *p*s < 0.032). PM performance differed significantly (*p* < 0.001) between the 2-word (81% correct) condition and the 6-word condition (58% correct). Performance in the 3-word condition (83% correct) differed significantly (all *p*s < 0.028) from performance in the 5-word (69% correct) and 6-word (58% correct) conditions. See Figure 1.

### Ongoing task

Data trimming was done separately for each block and each trial type for each participant. Several trials were excluded: (a) the initial five trials of Block 1 and Block 2; (b) trials that contained PM targets; (c) the three trials that followed a PM trial; (d) trials where RTs were greater than 2.5 SDs from a participant's grand mean; and (e) trials containing incorrect lexical decisions. Data trimming resulted in less than 4% of trials being eliminated.

#### Accuracy

Accuracy in the LDT was fairly high (percent of errors ranged from 2.6 to 4.3%) and did not differ by condition (*p* = 0.18) or by block (*p* = 0.28). See Table 1 for percent errors as a function of block and condition.

**Table 1:** **Percent errors in the lexical decision task as a function of block and condition**.

	***M***	***SD***
**BLOCK 1**		
Control	3.70	2.05
One word	2.56	1.82
Two word	3.63	1.72
Three word	2.48	2.01
Four word	3.10	2.78
Five word	2.47	1.56
Six word	4.34	3.87
**BLOCK 2**		
Control	2.92	2.25
One word	3.74	3.18
Two word	3.95	2.57
Three word	2.74	1.71
Four word	3.64	3.38
Five word	2.65	1.87
Six word	4.33	3.30

#### Reaction time costs

**Figure 2:**
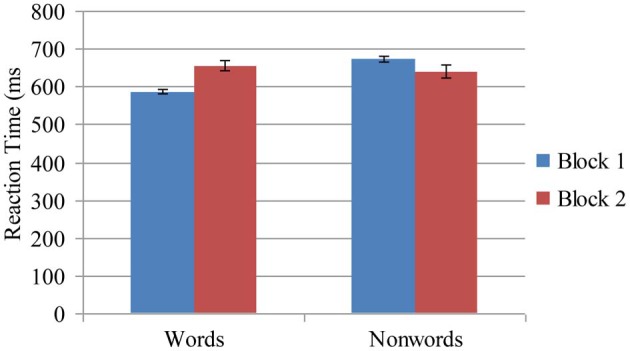
**Reaction time latencies measured in milliseconds as a function of lexical decision task word type (Words, Non-words) and as a function of block (Block 1, Block 2)**. Bars represent standard error.

**Figure 3:**
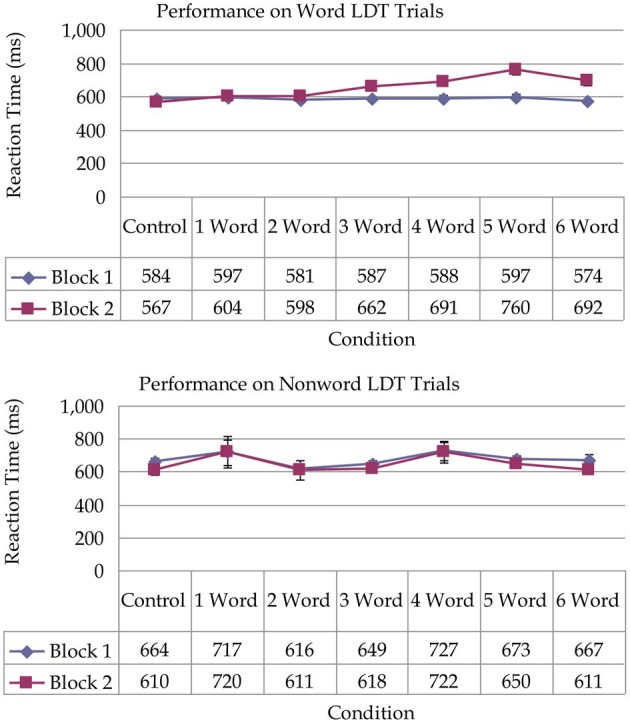
**Upper panel represents reaction time latencies measured in milliseconds on word trials in the lexical decision task as a function of condition**. Lower panel represents reaction time latencies measured in milliseconds on non-word trials in the lexical decision task as a function of condition. Bars represent standard error.

The average response time (RT) for word/non-word trials served as the dependent measure. We conducted a 2 (Word Type: word, non-word) × 2 (Block: block 1, block 2) × 7 (Condition: control, 1-word, 2-word, 3-word, 4-word, 5-word, 6-word) mixed factorial ANOVA with Word Type and Block as within-subject factors and Condition as a between-subjects factor. Results of this analysis revealed several significant effects. There was a main effect of Word Type, *F*_(1, 128)_ = 6.36, *p* < 0.05, η^2^ = 0.05 showing that reaction times were faster for word trials (*M* = 621 ms) compared to non-word trials (*M* = 657 ms). There was a significant main effect of block, *F*_(1, 128)_ = 8.66, *p* < 0.05, η^2^ = 0.06, revealing that performance was significantly slower in block 2 (*M* = 648 ms) compared to block 1 (*M* = 630 ms). There was no main effect of condition (*p* = 0.24). These main effects were qualified by several significant interactions. There was a significant Block × Word Type crossover interaction, *F*_(1, 128)_ = 85.00, *p* < 0.001, η^2^ = 0.40 showing that on word trials performance was significantly slower in block 2 compared to block 1. In contrast, for non-word trials, performance was faster in block 2 compared to block 1. See Figure 2. There was a significant 3-way interaction, *F*_(6, 128)_ = 7.36, *p* < 0.001, η^2^ = 0.26 which revealed a different pattern of responding across conditions for word and non-word trials. This interaction effect shows that reaction times increased linearly in block 2 for word trials (reflecting increasing costs) from the 2-word condition to the six-word condition, whereas performance on non-word trials did not reflect this pattern of increasing costs (see Figure 3).

## Discussion

In the current study, results replicated those of Cohen et al. (2008) and more powerfully demonstrated that attentional costs vary depending on the degree of cognitive load associated with a suspended intention and the relevance of ongoing task stimuli to the intention. In conditions in which cognitive load was high (3 or more intention-related targets), there were costs to ongoing task performance. It is important to note that the observed costs were material specific and only emerged on word trials in the LDT and not on non-word trials. Smallwood and Schooler (2006) claimed that much of the research on mind wandering had been influenced by a simple limited-capacity account of cognition, however, our data reflect a more nuanced pattern of responding. Participants were able to stay focused on the primary task when cognitive load was low and when stimuli were unrelated to the intention.

### Implications for our understanding of mind wandering

Mind wandering is thought to occur as a result of decoupled processing such that attention becomes coupled to internal thoughts and decoupled from perceptual information (Smallwood et al., 2003). However, it is important to understand what variables initiate the moment of decoupling from ongoing activity. It is difficult to ascertain from participants' self reports the precise moment that causes attention to be decoupled from a current task. In Smallwood (2013), the author states “At present, our inability to covertly detect the onset of self-generated mental activity is the central barrier to addressing the fundamental question raised by the phenomenon at both the theoretical and applied level” (p. 2). Results from the current study showed that disengaging from a primary task was more likely when (a) there was a high degree of cognitive load associated with a goal, and (b) when stimuli in the ongoing task were relevant to an unresolved goal. It is intuitive that goals associated with higher cognitive load influence the amount of mind wandering. It may be that the higher the cognitive load associated with a goal, then the more spreading activation that occurs to potentially associated cues in the environment. The current results also demonstrated that interference costs varied on a trial by trial basis depending on the relevance between the ongoing task stimulus and intention-related stimuli. That is, interference costs occurred solely on word trials in the LDT but not on non-word trials. Participants may have been unable to suppress intention-related thoughts due to the overlapping features between LDT words and the PM word targets and these intention-related thoughts were less likely to arise on non-word trials.

If mind wandering helps one to make progress toward a desired goal, then these lapses of attention can be viewed as instrumental. Given the frequency with which participants claim to engage in mind wandering, it seems unlikely that it would serve no functional role (Schooler et al., 2011). As mentioned previously, disengaging from a primary task in order to advance toward fulfilling a desired goal can be functional insofar as it helps to accomplish a desired future. Baird et al. (2011) provided a detailed analysis of the content of off-task thought which indicated that future oriented mind wandering usually involved a combination of self-relevant and goal-directed content. Baird et al. further suggested that the observed links between the self and future thinking in mind-wandering may be attributed in large part to the fact that prospective mind-wandering often involves planning for the future goals of the individual. For these reasons, interference costs in PM tasks can be thought of as a type of functional mind wandering because decoupling from the primary task serves the function of maintaining the intention in mind. In the current study, it is interesting to note that when speed on the ongoing task decreased in conditions of higher cognitive load, it was not accompanied by better PM detection. In fact, PM accuracy decreased as a function of condition. Results showed that the relationship between ongoing task performance and PM performance was not functional as ongoing task costs did not lead to better PM performance. The longer reaction times may reflect states of decoupled processing in which attention to the ongoing LDT was reduced in favor of internally generated thoughts. However, slowing in the LDT did not mean that it led to better PM performance. There are two possibilities to explain this finding. First, participants might have been more likely to decouple from the LDT when cognitive load was high but thoughts may have wandered to topics unrelated to the PM task. A second more likely possibility is that conditions with high cognitive load imposed such a difficult task requirement on participants that disengaging from the LDT did not necessarily lead to improved PM performance. For example, efforts to focus on internally generated thoughts about the PM task were in vain because due to task difficulty—it did not result in improved PM performance.

### Explanations for the pattern of increasing costs

An intriguing question is what accounted for the linear pattern of increasing costs. It might be that the overall intention came to mind on random trials and it took more time to cycle through intention-related cues in conditions of higher cognitive load. Alternatively, it might be that specific words in the LDT were perceived as semantically related to PM targets triggering thoughts of the intention. In this case, the higher the amount of intention-related cues, then the more frequently the intention would come to mind. It is impossible to answer this question with the existing data. However, it may be that the pattern of increasing costs occurred due to some or all of the following reasons: (a) it takes longer to cycle through the cues when there are more cues associated with an intention, and (b) when there are more cues associated with an intention, there are more possibilities that a LDT stimulus might overlap semantically with an intention word leading to more frequent attention shifts, and (c) participants might have weighed the PM task more heavily at the outset when there were more cues associated with the intention and therefore the intention came to mind more frequently. It is important for future research to arbitrate among these competing explanations. Future research paradigms could directly address this issue of what leads to decoupling from the ongoing task. One could design a task in which participants are given PM instructions similar to the current task. However, in one condition, there would be 12 words included in the LDT that were semantically related to the intention cue words. The interest would be in examining whether there would be more ongoing task costs in the condition with the 12 semantically related non-target words compared to the control condition. The results of this experiment might yield interesting data regarding whether shifts of attention from the ongoing task are initiated by specific words perceived to be related to the intention cues or whether shifts occur on a random basis.

Guynn's (2003) two-process model of strategic monitoring is useful when considering explanations for the current results. Guynn (2003) makes a distinction between two types of monitoring known as retrieval mode and item checking. When a person must execute a future intention it can be said that the cognitive system is in retrieval mode meaning that it is sensitive to the future possibility of an intention occurring. Retrieval mode can be thought of as similar to top-down strategies. When participants received the task instructions at the beginning of the task, they may have adopted a top-down attention allocation strategy in order to meet the demands of the ongoing LD task and PM task (e.g., Marsh et al., 2006). Presumably, high cognitive load led participants to weigh the PM task more heavily and allocate more resources to maintaining that intention in mind, thereby yielding more interference in the LD task. In line with this explanation, Horn et al. (2011) analyzed previous data from Smith (2003) using a cognitive process model known as the diffusion model (e.g., Ratcliff, 1978). Horn et al. (2011) interpreted their results to mean that including a PM task leads to more cautious speed-accuracy settings which results in higher latencies. These findings are similar to ideas expressed by Hicks et al. (2005) who suggested that strategic attention allocation policies determine how much attention will be devoted to the ongoing task and the PM task. However, if participants devoted more attention to the PM task in conditions of high cognitive load then one would expect that the pattern of increasing costs should have been exhibited for both word and non-word items. The material specific slowing in the current task implies that the second component of Guynn's model may be more useful as an explanation. The second component, item checking, involves post-stimulus checking for target events. Item checking can be thought of as similar to bottom-up strategies in which attention is devoted to the stimuli where the retrieval cue would be expected to occur. For each item, the participant must evaluate whether or not that stimulus is a retrieval cue for an intended action. In terms of the current results, a more probable explanation is that costs increased linearly due to participants having to cycle through more PM targets in conditions with higher numbers of targets. Item checking occurred on word trials as opposed to non-word trials because word trials provided a better match to the PM target items. It is worth noting that participants were surprisingly flexible in how they managed attention devoted to intention relevant stimuli (words) and intention irrelevant stimuli (non-words), even when these word/non-word stimuli occurred randomly on a trial by trial basis.

This sensitivity to goal-related information is reinforced by research by Klinger et al. who state: “… goal pursuit requires more than a passive memory of the pursuit; it requires a continuing state of sensitization to stimuli relevant to the pursuit and a readiness to act—to seize opportunities for attaining the goal even while not consciously thinking about it” (Klinger and Cox, 2011, p. 10). From a clinical perspective, cognitive theories of anxiety, and studies examining selective attention to threat, have emphasized the role of biases in attentional processes (e.g., Mogg et al., 2000). Cox et al. (2006) used an addiction-Stroop task to show that the strongest interference was found in participants who had strong current concerns (e.g., Klinger and Cox, 2011) about an addictive substance or in instances in which concerns were highlighted through experimental manipulations. As Cox et al. (2006) suggested, current concerns appear to serve as a motivational modulatory system leading to hypersensitivity to motivationally salient stimuli and sensitzing early perceptual pathways for analyzing structural features of input stimuli. Cox et al. continue to suggest that this causes automatic screening of environmental stimuli and functions as a general goal-lurking activity within the motivational system (p. 468). This account is very useful in helping to explain the current finding in which participants were able to screen out non-words and attend selectively to word stimuli.

### Replication of Cohen et al. (2008)

The lack of costs in the one-word and two-word conditions replicated findings from Cohen et al. (2008) and is in line with Einstein et al.'s (2005) multiprocess framework which suggest that intentions can be successfully executed in some instances with no observable costs to ongoing activity. In the Cohen et al. (2008) study, accuracy data in the LDT was not reported and Smith (2010) suggested that due to the absence of accuracy data, response times could not be clearly interpreted. Smith (2010) stated that the Block 1 baseline response times from Cohen et al. (2008) were rather high for the one-word condition. According to Smith (2010), this pattern suggested that Cohen et al.'s participants may have sacrificed speed for accuracy in the baseline block. However, the baseline response times in the current study are much more in line with previous research (e.g., Smith et al., 2007; Loft et al., 2008) and LDT accuracy did not differ from Block 1 to Block 2. Therefore, the absence of costs in conditions of low cognitive load reflects that an intention can be realized with no negative impact to ongoing task performance. It is rather puzzling why Cohen et al. (2008) and the current study both demonstrated no costs for the one-word condition which directly contradicts findings from Smith et al. (2007) and Smith (2010). The only explanation may be that there are subtle and nuanced differences in the way that the instructions are administered to participants which accounts for the different findings. It is hoped that future studies may help to illuminate what these differences may be.

As shown in Figure 1, PM performance decreased as the number of PM targets increased. In contrast to Cohen et al. (2008), in which there were no significant differences in PM accuracy across conditions, the current results revealed that PM performance varied significantly as a function of condition. The difference in results could be due to a lack of power in the Cohen et al. (2008) study, which had a fewer number of participants. The current finding that PM performance varied as a function of the number of targets is not surprising and is in line with previous research (e.g., Marsh et al., 2003; Einstein et al., 2005).

### Limitations and future research

It is important to note that although there are intriguing similarities between PM and mind wandering, there are also some differences. For example, the commonalities between PM and mind wandering only apply in cases when the contents of a mind wandering episode are unresolved goals and prospective in nature. Baird et al. (2011) showed that 48% of off-topic thought was future oriented, however, 52% involved thoughts about the present, past, and a small portion was devoted to thoughts with no temporal focus. Another difference between mind wandering and PM is that shifts away from a primary task in mind wandering tends to be spontaneous and unintentional. While attentional shifts in PM may at times be unintentional, they may also be deliberate and/or strategic. Furthermore, the mental contents during mind wandering episodes are often idiosyncratic to the individual. In PM paradigms, however, the contents often relate to the impending intention.

The contributions of this research represent the first preliminary steps toward better understanding the links between PM and mind wandering, however, it is important to note that they were of a speculative nature. There is a need for future research paradigms to disentangle the influences of unresolved goals and other forms of task-unrelated thoughts and their influence on ongoing task costs. Furthermore, future research should consider how the cognitive load associated with a goal influences mind wandering and what cues initiate decoupling from the ongoing task? The results from the current paper demonstrate that the higher the cognitive load associated with a PM intention, then the more attention shifts away from the ongoing task. One could imagine a comparable type of mind wandering scenario. For example, if I have six things that I need to do immediately following a meeting with a student, then it is more likely that my mind will wander to these impending tasks during the meeting compared to a day in which I may have only one thing to do following the meeting. In the latter situation, I might be more able to focus on the primary task of my meeting because there are fewer competing goals. To date, no studies on mind wandering have examined the number of cues greater than one associated with a goal, nor have they examined the relevance of primary task stimuli to unresolved goals.

An experiment is needed to examine whether increasing cognitive load associated with a suspended goal increases the likelihood that attention will be diverted away from ongoing activity toward that goal. Research by Goschke and Kuhl (1993) showed that information related to delayed intentions can be highly accessible, compared to information that is not future-oriented (i.e., the intention superiority effect). In their paradigm, participants were asked to memorize written descriptions of two activities, such as setting the table (“Spread the table cloth. Distribute the cutlery”) and clearing a messy desk (“Open the folder. Put in the files.”). Then, participants in an “execute” condition were informed that they would have to execute one of these scripts later, whereas those in an “observe” condition were instructed that they would later observe the experimenter carrying out one of the scripts. Results showed that correct recognition responses were faster for critical items related to the to-be-executed task compared to the to-be-observed task yielding an intention superiority effect. Interestingly, reaction times in this study on non-critical items were numerically slower in the execute conditions compared to the observation condition, although not reliable. However, this numerical difference could indicate that participants' thoughts during the recognition memory test may have been more likely to wander to the impending goal in the execute condition compared to a condition in which they did not have to execute the intention. The Goschke and Kuhl (1993) task could be easily adapted to investigate how cognitive load and the relevance of ongoing task stimuli influence reaction time latencies on non-critical items as an indication of mind wandering. For example, one could include execute conditions that had scripts with low, medium, or high cognitive load and also vary the number of script words that appeared in the recognition memory test. One would predict that reaction times on non-critical items would increase as a function of cognitive load.

## Conclusions

Our data suggest that the human cognitive system is configured in such a way that having an active goal does not impose equivalent costs on all ongoing activities. Rather, attentional costs vary depending on the degree of cognitive load associated with that goal and the degree to which the ongoing task stimuli are relevant to a goal. Most impressive is that attention costs varied on a trial by trial basis in the current paradigm even though participants could not possibly anticipate whether the next trial would be a word or non-word. Thus, on some proportion of word LDT trials, the appearance of a word stimulus might have led to thoughts along the lines of “this stimulus reminds me of something else I need to do ….” leading to longer reaction times. The pattern of increasing costs and the specificity of costs implies that even in cognitively demanding contexts, participants are sensitive to the properties of stimuli and how they relate to unresolved goals. This result is pertinent to mind wandering research because it suggests that the likelihood of mind wandering is greater when participants have goals with high cognitive load and when external stimuli are present that directly relate to an unfulfilled goal. Interference costs in PM paradigms may be an example of this special case of “functional mind wandering” (e.g., Schooler et al., 2011) in which disengaging from the ongoing task serves an important function of allocating attention to a delayed goal. However, as demonstrated in the current study, higher rates of disengaging from an ongoing activity do not necessarily ensure higher rates of goal attainment.

### Conflict of interest statement

The author declares that the research was conducted in the absence of any commercial or financial relationships that could be construed as a potential conflict of interest.

## References

[B1] Baird B. Smallwood J. Schooler J. W. (2011). Back to the future: autobiographical planning and the functionality of mind-wandering. Conscious. Cogn. 20, 1604–1611 10.1016/j.concog.2011.08.007 21917482

[B2] Cohen A.-L. Dixon R. A. Lindsay D. S. (2005). The intention interference effect and aging: similar magnitude of effects for young and old adults. Appl. Cogn. Psychol. 19, 1177–1197 10.1002/acp.1154

[B3] Cohen A.-L. Jaudas A. Gollwitzer P. M. (2008). Number of cues influence the cost of remembering to remember. Mem. Cogn. 36, 149–156 10.3758/MC.36.1.149 18323071

[B4] Cohen A.-L. Jaudas A. Hirschhorn E. Sobin Y. Gollwitzer P. M. (2012). Selective attention and prospective memory costs: the stimulus specific interference effect. Memory 20, 848–864 10.1080/09658211.2012.710637 22900905

[B5] Cohen A.-L. Kantner J. Dixon R. A. Lindsay D. S. (2011). The intention interference effect: the difficulty of ignoring what you intend to do. Exp. Psychol. 58, 425–433 10.1027/1618-3169/a000110 21592940 PMC4161040

[B6] Cox W. M. Fadardi J. S. Pothos E. M. (2006). The addiction–stroop test: theoretical considerations and procedural recommendations. Psychol. Bull. 132, 443–476 10.1037/0033-2909.132.3.443 16719569

[B7] Einstein G. O. McDaniel M. A. (1990). Normal aging and prospective memory. J. Exp. Psychol. Learn. Mem. Cogn. 16, 717–726 10.1037/0278-7393.16.4.717 2142956

[B8] Einstein G. O. McDaniel M. A. Thomas R. Mayfield S. Shank H. Morrisette N. (2005). Multiple processes in prospective memory retrieval: factors determining monitoring versus spontaneous retrieval. J. Exp. Psychol. Gen. 134, 327–342 10.1037/0096-3445.134.3.327 16131267

[B9] Gollwitzer P. M. (1993). Goal achievement: the role of intentions. Eur. Rev. Soc. Psychol. 4, 141–185 10.1080/14792779343000059

[B10] Gollwitzer P. M. (1999). Implementation intentions: strong effects of simple plans. Am. Psychol. 54, 493–503 10.1037/0003-066X.54.7.493

[B11] Goschke T. Kuhl J. (1993). The representation of intentions: persisting activation in memory. J. Exp. Psychol. Learn. Mem. Cogn. 19, 1211–1226 10.1037/0278-7393.19.5.1211

[B12] Guynn M. J. (2003). A two-process model of strategic monitoring in event-based prospective memory: activation/retrieval mode and checking. Int. J. Psychol. 38, 245–256 10.1080/0020759034400017822363699

[B13] Hicks J. L. Marsh R. L. Cook G. I. (2005). Task interference in time-based, event-based, and dual intention prospective memory conditions. J. Mem. Lang. 53, 430–444 10.1016/j.jml.2005.04.001

[B14] Horn S. S. Bayen U. J. Smith R. E. (2011). What can the diffusion model tell us about prospective memory? Can. J. Exp. Psychol. 65, 69–75 10.1037/a0022808 21443332 PMC3148193

[B15] Hunt R. R. Toth J. P. (1990). Perceptual identification, fragment completion, and free recall: concepts and data. J. Exp. Psychol. Learn. Mem. Cogn. 16, 282–290 10.1037/0278-7393.16.2.282 2137867

[B16] Klinger E. (1975). Consequences of commitment to and disengagement from incentives. Psychol. Rev. 82, 1–25 10.1037/h007617123501139

[B17] Klinger E. (1977). Meaning and Void: Inner Experience and the Incentives in People's Lives. Minneapolis, MN: University of Minnesota Press

[B19] Klinger E. (2009). Daydreaming and fantasizing: thought flow and motivation, in Handbook of Imagination and Mental Simulation, eds Markman K. D. Klein W. M. P. Suhr J. A. (New York, NY: Psychology Press), 225–239

[B20] Klinger E. Cox W. M. (2011). Motivation and the goal theory of current concerns, in Handbook of Motivational Counseling, 2nd Edn, eds Cox W. M. Klinger E. (Chichester: Wiley), 3–47

[B21] Kucera H. Francis W. N. (1967). Computational Analysis of Present-Day American English. Providence, RI: Brown University Press

[B22] Loft S. Kearney R. Remington R. (2008). Is task interference in event-based prospective memory dependent on cue presentation? Mem. Cogn. 36, 139–148 10.3758/MC.36.1.139 18323070

[B23] Marsh R. L. Cook G. I. Hicks J. L. (2006). Task interference from event-based intentions can bematerial specific. Mem. Cogn. 34, 1636–1643 10.3758/BF03195926 17489290

[B24] Marsh R. L. Hicks J. L. Bink M. L. (1998). Activation of completed, uncompleted, and partially completed intentions. J. Exp. Psychol. Learn. Mem. Cogn. 24, 350–361 10.1037/0278-7393.24.2.350

[B25] Marsh R. L. Hicks J. L. Cook G. I. Hansen J. S. Pallos A. L. (2003). Interference to ongoing activities covaries with the characteristicsof an event-based intention. J. Exp. Psychol. Learn. Mem. Cogn. 29, 861–870 10.1037/0278-7393.29.5.861 14516219

[B26] Mogg K. McNamara J. Powys M. Rawlinson H. Seiffer A. Bradley B. P. (2000). Selective attention to threat: a test of two cognitive models of anxiety. Cogn. Emot. 14, 375–399 10.1080/026999300378888

[B27] Ratcliff R. (1978). A theory of memory retrieval. Psychol. Rev. 85, 59–108 10.1037/0033-295X.85.2.59

[B28] Schooler J. W. Reichle E. D. Halpern D. V. (2004). Zoning out while reading: evidence for dissociations between experience and metaconsciousness, in Thinking and Seeing: Visual Metacognition in Adults and Children, ed Levin D. T. (Cambridge, MA: MIT Press), 203–226

[B29] Schooler J. W. Smallwood J. Christoff K. Handy T. C. Reichle E. D. Sayette M. A. (2011). Meta-awareness, perceptual decoupling and the wandering mind. Trends Cogn. Sci. 15, 319–326 10.1016/j.tics.2011.05.006 21684189

[B30] Scullin M. K. Einstein G. O. McDaniel M. A. (2009). Evidence for spontaneous retrieval of suspended but not finished prospective memories. Mem. Cogn. 37, 425–433 10.3758/MC.37.4.425 19460950

[B31] Smallwood J. (2011). Mind-wandering while reading: attentional decoupling, mindless reading and the cascade model of inattention. Lang. Linguist. Compass 5, 63–77 10.1111/j.1749-818X.2010.00263.x

[B32] Smallwood J. (2013). Distinguishing how from why the mind wanders: a process–occurrence framework for self-generated mental activity. Psychol. Bull. 139, 519–535 10.1037/a0030010 23607430

[B33] Smallwood J. Baraciaia S. F. Lowe M. Obonsawin M. C. (2003). Task unrelated thought whilst encoding information. Conscious. Cogn. 12, 452–484 10.1016/S1053-8100(03)00018-7 12941287

[B34] Smallwood J. McSpadden M. C. Schooler J. W. (2008). When attention matters: the curious incident of the wandering mind. Mem. Cogn. 36, 1144–1150 10.3758/MC.36.6.1144 18927032

[B35] Smallwood J. Schooler J. (2006). The restless mind. Psychol. Bull. 132, 946–958 10.1037/0033-2909.132.6.946 17073528

[B36] Smith R. E. (2003). The cost of remembering to remember in eventbased prospective memory: investigating the capacity demands of delayed intention performance. J. Exp. Psychol. Learn. Mem. Cogn. 29, 347–361 10.1037/0278-7393.29.3.347 12776746

[B37] Smith R. E. (2010). What costs do reveal and moving beyond the cost debate: reply to Einstein and McDaniel (2010). J. Exp. Psychol. Learn. Mem. Cogn. 36, 1089–1095 10.1037/a0019183 20852726 PMC2940056

[B39] Smith R. E. Hunt R. R. McVay J. C. McConnell M. D. (2007). The cost of event-based prospective memory: salient target events. J. Exp. Psychol. Learn. Mem. Cogn. 33, 734–746 10.1037/0278-7393.33.4.734 17576150

